# Satisfaction Level of Tuberculosis Patients Regarding Their Access to TB Care and Prevention Services, Delivered Through a Public–Private Mix Model in Pakistan

**DOI:** 10.3390/healthcare7040119

**Published:** 2019-10-18

**Authors:** Syed Mustafa Ali, Naveed Anjum, Farah Naureen, Aamna Rashid, Adeel Tahir, Muhammad Ishaq, Muhammad Usman

**Affiliations:** 1 Monitoring, Evaluation and Learning Unit, Mercy Corps, Lane 9, Chak Shehzad, Park Road, Islamabad 44000, Pakistan; 2 Pakistan Scientific and Technological Information Center (PASTIC), Quaid-e-Azam University (QAU) Campus, Islamabad 44000, Pakistan

**Keywords:** patient satisfaction, quality of care, private healthcare provider, public–private mix model, tuberculosis control, health system

## Abstract

*Objective*: The private healthcare providers (PHCP) are believed to improve access to healthcare services in public–private mix (PPM) projects, as they are considered first point of contact for healthcare. The purpose of this study was to determine the satisfaction level of tuberculosis (TB) patients. *Design*: A questionnaire-based, cross-sectional study was carried out during November and December 2017 for 572 under-treatment patients registered with PHCPs in the PPM project. Lot quality assurance sampling technique was used to randomly select 19 districts from a sample frame of 75 districts. In each selected district, the data collector retrieved a TB register of 8 months (January–August 2017) and systematically selected patients by fixed periodic interval. SPSS version 24.0 (IBM Corp, Amonk, NY, USA) was used to analyze the data. *Results*: This study included 53% (*n* = 301) males and 47% (*n* = 271) females, with mean age of 38 years (SD, ±18). Almost half of the participants were illiterate (51%, *n* = 289), and 64% (*n* = 365) were non-earning members of the family. In practice, most of the participants visit private providers (71%, *n* = 407), including private hospitals/clinics (44%) and traditional practitioners (27%; *n* = 153); 55% of participants visited their current doctor because of the clinic’s proximity to their residence. Of the participants, 82% (*n* = 469) were satisfied with TB care services and 85% (*n* = 488) said that they would recommend this clinic to others. *Conclusion*: PHCPs are preferred providers for individuals, which is consistent with findings of other studies. Though they are satisfied with TB care and services, interventions should be introduced to reduce the financial burden on the patient. Partnering PHCP is a way forward to ensure universal health coverage and better health outcomes of the population.

## 1. Introduction

In response to The End TB (tuberculosis) Strategy, countries should place patients at the center of the care delivery system by adopting innovative and cost-effective approaches. It is essential to advance the fight against TB by promoting access and utilization of healthcare services, so that the goal of ending TB by 2030 is achieved [1].

The perception of the patient about his/her own health status is a determining factor for the patient’s treatment adherence and treatment outcome [2]. Additionally, evidence suggests that public–private mix (PPM) interventions have achieved increased case notification, treatment outcomes, and patient satisfaction [3,4].

The link between treatment adherence and patient satisfaction level is already established, and it is demonstrated that dissatisfied patients may become non-compliant to treatment protocols or miss their consultation appointments [5,6]. On the contrary, satisfied patients are less likely to encounter challenges in gaining access to healthcare services [7]. Therefore, focus of medical attention is shifted from illness-centered care to patient-centered care [8].

Private healthcare providers (PHCPs) are believed to improve access to healthcare services, and in many of these PPM projects, PHCPs are considered a first point of contact for healthcare [9]. The PHCPs constitute the biggest part of the healthcare system. However, cost of care is higher for private doctors, whereas it is lower at the clinics enrolled with non-governmental or welfare organizations [10,11,12].

Patient satisfaction surveys are planned to examine patients’ needs and expectations by collecting their opinions on healthcare services and their delivery [13]. Patient satisfaction is the perceived gratification of the patient’s needs and desires in response to healthcare services [14]. Exploring a patient’s satisfaction level is helpful in assessing responsiveness of the healthcare system [15], which is an important part of said healthcare system. It also helps in understanding how well a patient’s needs are met and helps in assessing process of care [15].

Improving access to and utilization of healthcare services is pivoted upon accessibility, acceptability, and affordability. Accessibility refers to individual’s recognition of need for the healthcare services and their willingness to utilize those services. However, access to healthcare services is influenced by social, cultural, and environmental factors [16]. Affordability determines if a person has adequate resources to pay for costs related to healthcare services [17]. However, acceptability is a poorly defined dimension of access. With inconsistencies found in the literature, it can be described as a patient’s perception about his/her participation in an intervention and about the perceived or actual effectiveness of the intervention [18]. However, it is argued that perception about acceptability may likely change with actual experience of the intervention [19].

The purpose of conducting patient satisfaction surveys and appraising TB patients’ access to healthcare services is a cornerstone of the efforts for improving quality of care. Therefore, apprising patients’ access to healthcare services by utilizing three dimensions of access, accessibility, acceptability and affordability—is the main objective of this study (See Supplementary Information).

## 2. Materials and Methods

### 2.1. Aim of the Study

The aim of the study was to examine the satisfaction level of tuberculosis patients regarding access to TB care and prevention services, delivered through PPM-enrolled private healthcare providers (PHCPs). The study was planned and executed as part of the End of Project (EoP) Evaluation of New Funding Mechanism (NFM) of Public–Private Mix Model, funded by Global Fund and implemented by Mercy Corps Pakistan, along with its partners.

### 2.2. Study Settings

As part of Pakistan’s National TB Control Programme, Mercy Corps Pakistan implemented an NFM from July 2015 to December 2017 in 75 districts of Pakistan, along with its 7 implementation partners. PPM-enrolled PHCPs were given financial incentive to support their consultation time during the course of treatment. Anti-TB drugs, diagnostic services, and consultation were provided free-of-cost once a patient registered as a TB case, regardless of case category or type.

### 2.3. Study Design

A population-based, cross-sectional survey was carried out by using a quantitative method of data collection from November to December 2017.

### 2.4. Sampling Strategy

A two-stage sampling method was used to identify and recruit the study participants.

#### 2.4.1. Sampling of District

At the first stage, proportional stratified random sampling was conducted to select a lot of 19 districts (using lot quality assurance sampling technique) from a sample frame of 75 districts, where the NFM’s PPM intervention was implemented. Five strata were developed, which are commonly used in other national-level surveys, for the purpose of sampling, including Punjab, Sindh, Khyber Pakhtunkhwa (KPK), Balochistan, and others (Islamabad Capital territory, Azad and Jammu Kashmir (AJK), Gilgit Baltistan (GB)). Implementation districts were organized into strata and sampled according to the respective weight of population districts. District names were organized into stratum and randomization was done in Microsoft Excel, using the randomization function. Sampling results are given below in Table 1.

#### 2.4.2. Sampling of Participants

At the second stage, sample size was calculated by using an online tool (http://www.raosoft.com/samplesize.html). For this purpose, population size was taken from notification data reported for July 2015–August 2017, i.e., 72,183 all type cases. By keeping the confidence level at 95% and the confidence interval (margin of error) at 4, the recommended sample size was 596.

The sample size was achieved from the notification record of 8 months, i.e., January 2017 to August 2017. This cohort of the notified cases included those who were registered for treatment during these 8 months, hence we recruited cases across the spectrum of treatment timeline (initiation to end). Notification record of the period June 2015 to December 2016 was not included because of the possible difficulty in reaching those patients who had completed their treatment. The recommended sample size was distributed proportionately among 19 districts, i.e., districts with more case notifications had a sample size proportional to its size. The District TB Register (TB03) of said period of 19 selected districts was used for enlisting patients, and potential respondents were selected systematically from the list, e.g., every *n*-th number of the listed patients. Randomization number was allocated to each district by the lead researcher to avoid any potential bias of the data collector.

### 2.5. Recruitment of Participants

In each selected district, the data collector retrieved the TB03 record of 8 months and prepared a list of systematically selected patients. After this, patients’ names were organized into clusters based on their geographical location, as mentioned in the TB03 record. Patients with incomplete contact details (contact number and address) were excluded from the list of selected patients. All patients were contacted telephonically (if contact number was given) before visiting them, and informed consent was sought.

### 2.6. Data Collection

The study used a “patient satisfaction survey tool” that aimed to appraise the patient’s access to healthcare services. In terms of utilization of services, access was measured through accessibility, affordability, and acceptability, as proposed by Gulliford et al. [16]. Study-adapted satisfaction-related variables from validated tools [20,21,22] and modification were included to make them fit-for-context. The data collection tool was translated into the local language (i.e., Urdu) and collected using Open Data Kit (ODK) Collect (an open source suite of tool: http://opendatakit.org).

The project evaluation team (F.N., S.M.A., N.A.) developed the patient satisfaction measurement tool and imparted training to data collectors on data collection and transfer. The training session included the objective of study, the underlying theoretical concepts, the orientation on the tool, and role play. The tool was pre-tested before its actual use.

### 2.7. Data Analysis

The data sheet was imported into SPSS version 24.0 (IBM Corp, Amonk, NY, USA) and data were checked for anomalies; missing data were treated accordingly. Basic descriptive analyses were performed, and results are presented in the form of frequencies, percentage, mean, and standard deviation.

### 2.8. Ethics Approval

Before commencement of the study, ethical approval was sought from the ethical review committee of the International Research Force, Pakistan. The reference number of the ethical approval is IRFIRB092017/MC02.

## 3. Results

### 3.1. Overview of Participants

Due to data completeness issues, combined with security concerns in two of the selected districts, we achieved a 572 sample, instead of the desired sample size of 596. Sample population included 53% (*n* = 301) males and 47% (*n* = 271) females, with mean age of 38 years (SD, ±18). Of the male participants, 65% (*n* = 197) were earning in different capacities (business, job etc.), 26% (*n* = 78) were unemployed, 9% (*n* = 26) were students, and 58% (*n* = 175) were educated up to or less than class 5. Similarly, 82% (*n* = 224) of the recruited females were housewives, 14% (*n* = 37) were students, and 73% (*n* = 197) were educated up to or less than class 5.

Overall, almost half of the recruited population was illiterate (51%, *n* = 289), and 64% (*n* = 365) of them were non-earning. Of the recruits, 45% (*n* = 256) were bacteriologically-confirmed and 55% were clinically diagnosed cases, including 42% (*n* = 242) of pulmonary cases and 13% (*n* = 74) of extra-pulmonary cases. Moreover, 93% of them (*n* = 533) were category-I (Cat-I) patients, and the remainder (7%, *n* = 39) were category-II (Cat-II) patients (Table 2).

### 3.2. Accessibility

#### 3.2.1. Accessing Healthcare Services

Generally, for health-seeking, most of the participants visited private providers (71%, *n* = 407), including 44% that visited private hospitals/clinics and 27% (*n* = 153) that visited traditional practitioners (herbalist, spiritual healer, etc.). However, 29% of respondents visited government hospitals for seeking healthcare services. A slightly higher percentage of females (74%; 200/271) visited private facilities for seeking healthcare services, compared to males (69%; 207/301) that visited private healthcare facilities. More than half of the participants (*n* = 314, 55%) visited a presently serving PHCP because it was the closest service provider.

Survey participants also provided reasons for the selection of healthcare facility type (public or private). ‘Saves time’ and ‘close to residence’ were the frequently reported reasons, regardless of participants’ selection of healthcare facility type. Of the participants, 173 (out of 407; 42%) provided ‘close to residence’ as a reason to access private healthcare facilities, as opposed to 52 participants (out of 165; 31%) who preferred to visit government hospitals. Those who preferred to visit private clinics/hospitals (*n* = 254) reported ‘good reputation of doctor’ and ‘past treatment experience’ as major reasons for consulting PPM-enrolled PHCPs. Only 14 participants decided to visit PPM-enrolled PHCPs, as they were informed that TB treatment services are free of cost here.

It is also noted that participants’ preferred choice of provider was different from their general health-seeking practice. For example, 27% of the participants (*n* = 153) visited traditional practitioners for seeking healthcare services, whereas only 5% of participants (*n* = 27) actually preferred them for seeking healthcare services. A vast majority of participants (73%; 419 out of 572) preferred to seek medical services from a general practitioner or doctor.

It is reported that the waiting time to consult a doctor was more than the waiting time to collect medicine from the clinic staff. Of the participants, 36% (*n* = 206) waited less than 15 min to receive consultation; however, 72% of participants (*n* = 414) collected medicine in same time after consultation. Of the participants, 17% (*n* = 99) waited more than 45 min to receive consultation from a doctor. Surprisingly, there were 5% more females (20% female, 55/271; 15% males, 44/301) who waited for more than 45 min to receive consultation.

#### 3.2.2. Access to Information

Currently, a doctor is a primary source to share information concerning different aspects of TB care and prevention. A majority of patients, regardless of age, sex, and education, reported that they received information about treatment duration (94%, *n* = 540) and significance of taking regular medicine (90%, *n* = 514). One fourth of the participants (*n* = 146) claimed that they were aware about TB symptoms and its treatment before they were diagnosed with TB. Almost half of the participants (49%, *n* = 283) were told about the possible side or unwanted effects of the anti-TB drugs; however, 42% of them (*n* = 240) were informed about the management response of the PHCP when they experienced any side effects. Almost 86% of the participants were aware of their correct treatment duration, i.e., either 6 months or 8 months, whereas the remainder reported different treatment durations.

#### 3.2.3. Access to Patient

Of the participants, 67% (*n* = 384) said that they will allow anyone to contact them or visit their home for treatment reminders and other treatment-related support. On the contrary, 28% of the participants (*n* = 162) said that they will not allow any contact or visitor. Additionally, 60% of the participants informed that they were not contacted by anyone regarding their TB treatment. In regard to bacteriologically-confirmed TB cases (*n* = 256), it was observed that only 57% of these cases (*n* = 147) were contacted during the treatment period for any purpose.

### 3.3. Affordability

#### 3.3.1. Care-Related Responsibility and Cost of Treatment

Over one fourth of the participants (27%; *n* = 158) claimed that they were responsible for their own treatment-related expenses; 66% of the participants mentioned their dependence on the male member of their family, including husband (*n* = 132, 23%), father (*n* = 112, 20%), son (*n* = 86, 15%), and brother (*n* = 45, 8%). There were only 12 participants who were dependent on their mother for their treatment-related expenses.

The mean cost for transportation was 306 PKR (Pakistani Rupees; 150 PKR=1.0 USD) for a round trip between the clinic and residence; however, median cost was 100 PKR. An independent sample *t*-test was conducted to compare mean transportation costs between males and females. There was a significant difference in the mean transportation costs between male (M = 160, SD = 267) and female (M = 467, SD = 1526) conditions; t (285) = −3.26, *p* = 0.001. The result suggests that females spend more on transportation costs compared to males, which can potentially limit the access of females to PHCP facilities.

#### 3.3.2. Livelihood Challenges

A significant proportion of the survey participants (65%) reported no livelihood challenge. However, 35% of the participants (*n* = 201) reported different types of challenges related to their work or education. Of the participants, 61 (30%, 61/201) said that they had to work less hours which affected their earning potential, hence affected income, and 31% of the participants (*n* = 62) reported that they had to take leave work, while 52 participants quit their jobs because of the disease. Out of the 63 students, 17 students (27%) reported that they had to either quit studies or it affected their education badly.

### 3.4. Acceptability

#### 3.4.1. Responsiveness of Healthcare Provider

The majority of the participants agreed that their doctor was respectful to them (*n* = 484, 84%) and listened to them whenever they had any complaints (*n* = 485, 85%). In addition, they also agreed that the paramedic was respectful to them (*n* = 485, 85%).

#### 3.4.2. Patient Satisfaction

Overall, 82% of the participants (*n* = 469) were satisfied with TB care services. The correlation between the patients’ perception about adequacy of consultation time and their satisfaction level was found to be statistically significant at 0.01 level (2-tailed), r = 0.789, *n* = 572, *p* = 0.00. However, as suggested by a chi-square test for independence of variables (at significance levels of 0.01 and 0.05), there was no relationship between participants’ satisfaction level and their level of education and occupation. Of the participants, 83% were satisfied with the time their doctor had spent on him/her for consultation, and 66% of the survey participants said that they are likely to visit the same clinic in case of any other healthcare problems. A significant proportion of survey participants (85%, *n* = 488) said that they will recommend this clinic to others.

#### 3.4.3. Patients’ Privacy and Confidentiality

Of the participants, 60% said that they would feel bad if their identity as a tuberculosis patient was disclosed in the community. A chi-square test of independence was conducted to compare the frequency of such cases in males and females. It was found that this feeling about disclosure of their identity as a TB patient is independent of gender (χ2 (2) = 1.66, *p* = 0.44). A significant proportion of the survey participants (79%) perceived that their personal information was kept secure at the clinic, and only 1% of the participants had doubt about the security of the personal information.

Only 15% of the participants said that they would have a problem in sharing their contact details, including contact number and home address. Of the participants, 80% (303 out of 384) who allowed anyone to contact them or visit their home for reminders and support purposes also said that they would not have a problem sharing their contact details, including contact number and home address.

## 4. Discussion

The study measured patient satisfaction levels upon three aspects of the quality of care, namely, accessibility, affordability, and acceptability. To our knowledge, it is the first study to measure the satisfaction level of TB cases who consult private healthcare providers for tuberculosis prevention and care services in Pakistan. A link between satisfied patients and positive treatment outcome is already established [23,24], hence TB eradication efforts should include mechanisms to record patient satisfaction with TB care and prevention services [24].

The results show that individuals’ preferred choice of healthcare provider (doctor) was different from the provider type they visit in practice (informal providers). Most of them consult private healthcare providers (both formal medical practitioners and traditional or informal providers) in the case of any ailment. Almost 80% of the patients contact private healthcare systems in case of illness, due to unavailability of public healthcare professionals. In addition, belittling of private healthcare providers or systems is gradually fading away [25], as it has become a long standing fact that most of the healthcare services are delivered through the private healthcare system [26,27]. Therefore, establishing public–private partnership for health system strengthening is integral in sustaining long-term positive effects on the health status of people [28].

Despite free availability of TB drugs, some persons with tuberculosis have incurred costs in getting medicine. Elsewhere in the world, where TB drugs are provided free of charge, the same situation has been reported [28,29]. Given the poor nutritional status of persons with tuberculosis, doctors prescribe additional supplements, for which patients have to pay out of their pockets. In cases where additional medication or supplements are warranted, programs should aim to provide them free of cost [29]. Nonetheless, better nutrition, along with other factors (housing, habitat, hygiene, sanitation), are attributed to decreased tuberculosis notifications [30]. In addition, loss of job and reduced income add financial hardships to a person affected with tuberculosis [29]. Therefore, financial support schemes (e.g., insurance) and social support mechanisms can be introduced to reduce economic burden, with careful ways of governing such initiatives [31].

Our study showed that 82% of the participants (*n* = 469) were satisfied with the TB care services delivered at the private healthcare facilities. However, measures of patient satisfaction are related to several indicators, including physical infrastructure, behavior of providers, availability of medicine, emotional support, cost of service, and respect of patients’ choices [32]. Our study showed positive response on almost all of these indicators that somehow explain patients’ high satisfaction levels with services. On the contrary, there are studies showing low satisfaction level of patients who have received services from public healthcare facilities [33,34,35].

## 5. Conclusions

Interdependence between patients’ satisfaction and healthcare planning is inevitable for developing responsive healthcare systems. In Pakistan, like other developing countries, the private healthcare sector is an integral constituent of the healthcare system. Consistent with the findings of previous studies, private healthcare providers are the preferred choice for most individuals. Therefore, healthcare agencies or regulators should give due attention to the private healthcare sector, so that response to serious health conditions like tuberculosis can be prepared and services delivered in a coordinated manner. Partnering with PHCPs is a way forward to ensure universal health coverage and better health outcomes of the population. Additionally, it is also important to acknowledge the presence of informal healthcare providers. Therefore, a national-level dialogue for their engagement and regulation should be initiated to strengthen the healthcare delivery system. A patient satisfaction survey is an essential tool to assess the responsiveness of the system by capturing patients’ expectations. Planning of healthcare services, inclusive of people’s needs, can subsequently reduce economic burden of illness and can improve health outcomes and wellness of people.

## Figures and Tables

**Table 1:** Sampling of district.

	Total Districts in Stratum	Weighted % Age	Sampling	Sample Districts
Punjab	27	36%	7	Rajanpur
				Sahiwal
				Jhang
				Pakpattan
				Bhakkar
				Narowal
				Sialkot
Sindh	16	22%	4	Badin
				Larkana
				Umerkot
				Khairpur
KPK	15	20%	4	Malakand
				Swat
				Nowshehra
				Charsadda
Balochistan	9	12%	2	Quetta
				Lasbela
AJK, GB, and Islamabad	8	10%	2	Islamabad
				Bhimber
Total Districts (*n*)	75	100%	19	

Whereas KPK—Khyber Pakhtunkhwa; AJK—Azad and Jammu Kashmir; GB—Gilgit Baltistan.

**Table 2:** Overview of the characteristics of the study participants.

Characteristics	Variables	Number (%)
Sex	Male	301 (53)
	Female	271 (47)
Age	15–24	180 (31)
	25–34	101 (18)
	35–44	75 (13)
	45–54	78 (14)
	55–64	71 (12)
	More than 65	67 (12)
Mean Age	Years (SD)	38 (±18)
Level of Education	Illiterate	289 (51)
	1–5 grade	86 (15)
	6–8 grade	58 (10)
	9 and 10 grade	76 (13)
	11–14 grade	55 (10)
	15 and 16 grade	8 (1)
Occupation	Unemployed	78 (14)
	Housewife (unemployed)	224 (39)
	Student (unemployed)	63 (11)
	Farmer	41 (7)
	Labor	77 (13)
	Small Business	44 (8)
	Govt. employee	9 (2)
	Private employee	36 (6)
Disease Site	Pulmonary (Bac+)	256 (45)
	Pulmonary (clinically-diagnosed)	242 (42)
	Extra-pulmonary (clinically-diagnosed)	74 (13)
Treatment Category	Cat–I	533 (93)
	Cat–II	39 (7)

Whereas SD—Standard Deviation; Bac+—Bacteriologically confirmed; Cat—Category.

## Supplementary Materials

The following are available online at https://www.mdpi.com/2227-9032/7/4/119/s1: Supplementary Information: End of Project Evaluation–New Funding Modela Patient satisfaction survey.
