# Supplementary material for: Satisfaction Level of Tuberculosis Patients Regarding Their Access to TB Care and Prevention Services, Delivered Through a Public–Private Mix Model in Pakistan
Source: Healthcare (Basel). 2019 Oct 18;7(4):119. doi: 10.3390/healthcare7040119 (PMC6955770; doi:10.3390/healthcare7040119)
Supplement: Supplementary file 1 [file healthcare-07-00119-s001.pdf]

End of Project Evaluation – New Funding Model

Patient Satisfaction Survey

Domains: (1) Perspective on Quality of Care, (2) Access to Information, (3) Access to Services, (4) Responsiveness, (5) Affordability of Services, (6) Case Management

Sub-Study 3:

Understanding patient's perspective and experience to appraise the access to healthcare services

Date		District	
Patient Name		Gender	1. Male<br>2. Female
Age		Education in years (completed years)	
Occupation		Contact Number	
Disease site	1. Pulmonary<br>2. Extra Pulmonary	Treatment category	1. Cat -1<br>2. Cat -2
Interviewer Name			

Accessibility (Service and information) Measurement Scale		
1.	Please tell us about your education and occupation?	
2.	Usually where do you and your family go for treatment?	1. Government Hospital<br>2. Private clinic/hospital<br>3. Other (traditional healers, hakeem,)
3.	Reason for Preference (Multiple selection is allowed)<br>1. Saves time (waiting time)<br>2. Saves money<br>3. Staff is more competent<br>4. Is close to the residence<br>5. Trust on the healthcare provider<br>6. Good relationship with healthcare provider or any of the staff members<br>7. Provider has a good behavior (polite etc)<br>8. Provider gives adequate time for consultation or medical attention	
4.	If you prefer a private doctor/clinic, why did you select this private healthcare facility (Multiple selection is allowed)<br>1. Someone referred you	

	2. You were referred from a chest camp<br>3. Because of free TB treatment<br>4. Doctor's good reputation<br>5. Past treatment experience<br>6. Good behaviour of doctor<br>7. Other (please describe)___________________________________	
5.	Is this doctor/clinic the closest doctor/clinic from your house?	1. Yes<br>2. No<br>3. May be
6.	Which level of provider you would prefer to see first?	1. Traditional practitioners (Hakeem, Homeo etc)<br>2. Mid-level Providers (dispenser, LHV, Health Tech)<br>3. Doctor/GPs
**Note: Please select one number from given range, where 1 is the best/always and 5 is the worst/never**		
7.	The paramedic (clinic staff) was available whenever you needed him (Babikako 2011)	① ② ③ ④ ⑤
8.	The doctor spent enough time consulting and diagnosing TB?	① ② ③ ④ ⑤
9.	Were you informed about the duration for which you will need to continue taking anti-TB drugs? Mention period?	1. Yes ____________<br>2. No<br>99. Don't Know
10.	Were you informed about the significance of taking regular medicine?	1. Yes<br>2. No<br>99. Don't Know
11.	Were you informed if taking medicine would have unwanted effects or side effects? (Babikako 2011)	1. Yes<br>2. No<br>99. Don't Know
12.	Were you informed about what should you do if you experience any unwanted side effects? (Babikako 2011)	1. Yes<br>2. No<br>99. Don't Know
13.	How long did you wait for getting results of lab tests?	1. <15 minutes<br>2. 16-30 minutes<br>3. 31-45 minutes<br>4. >45 minutes
14.	How long did you have to wait for receiving treatment from GP? (waiting time)	1. <15 minutes<br>2. 16-30 minutes<br>3. 31-45 minutes<br>4. >45 minutes
15.	How long did you have to wait for collecting medicine?	1. <15 minutes<br>2. 16-30 minutes<br>3. 31-45 minutes<br>4. >45 minutes
16.	Do/did you allow anyone to contact you or visit your home for reminder or other support?	1. Yes<br>2. No<br>99. Don't Know
17.	During your treatment, did someone contact you for reminder, sharing treatment related information? Please explain (who? how many times? for what purpose)	

18.	Did the provider give you information about how spread of TB can be prevented?	1. Yes<br>2. No<br>99. Don't Know
19.	Before you got diagnosed, were you aware about TB disease, its symptoms, prevention and treatment?	1. Yes<br>2. No<br>99. Don't Know
**Affordability Measurement Scale**		
20.	Who is responsible for your TB related treatment related expenses?	1. Yourself<br>2. Father<br>3. Husband<br>4. Brother<br>5. Other: ___________
21.	How much did you spend on treatment (consultation, labs and drugs) before you were diagnosed for TB?	Amount________
22.	How much have you spent on treatment (consultation, labs and drugs) after being diagnosed for TB?	
23.	After you were diagnosed with TB, did you pay for the following services? If yes, please mention amount	1. TB medicine (Rs____)<br>2. Doctor consultation (Rs____)<br>3. Lab test (Rs____)
24.	Generally, how much cost have you incurred in visiting this clinic for TB treatment? (round trip)	Amount_________
25.	During treatment, did you face any livelihood challenge (e.g., off-days, job termination, salary deduction, effect on daily wage), if yes probe about the detail.	
26.	What is your opinion about the cost of TB treatment?	
**Acceptability Measurement Scale**<br>Note: Please select one number from given range, 1 be the best/always and 5 be the worst/never		
27.	Doctor listened to me fully, whenever I had any complaint	① ② ③ ④ ⑤
28.	The paramedic was respectful towards me	① ② ③ ④ ⑤
29.	The doctor was respectful to me	① ② ③ ④ ⑤
30.	How satisfied are you with the overall care and services received at this clinic? (Babikako 2011)	① ② ③ ④ ⑤
31.	How satisfied were you with amount of time the doctor(s) spent with you during clinic visit? (Babikako 2011)	① ② ③ ④ ⑤

32.	Doctor or staff of other hospital/clinic (which you visited on previous occasion) treated you in efficient and better way? (Babikako 2011)	① ② ③ ④ ⑤
33.	How would you feel if your identity is disclosed as TB patient in community?	1. Good<br>2. Bad<br>3. Doesn't matter
34.	Would you recommend this clinic to somebody else for seeking healthcare? (Babikako 2011)	1. Yes<br>2. No<br>3. May be
35.	If you had another illness again, would you choose the same clinic/doctor? (Babikako 2011)	1. Yes<br>2. No<br>3. May be
36.	The results of the TB treatment will be as good as you expect? (Babikako 2011)	1. Yes<br>2. No<br>3. May be
37.	Do you think your personal information is kept secure in clinic?	1. Yes<br>2. No<br>3. May be
38.	Did you receive drugs on time (at clinic) without being asked for wait of couple of days?	1. Yes<br>2. No<br>3. May be
39.	Do you have any problem in sharing your contact details (contact number, home address)	1. Yes<br>2. No<br>3. May be
40.	Before starting treatment, were you aware that medicine of TB are provided free of cost?	1. Yes<br>2. No
